# A Novel Approach to Minimising Acute Equine Endometritis That May Help to Prevent the Development of the Chronic State

**DOI:** 10.3389/fvets.2021.799619

**Published:** 2022-01-06

**Authors:** J. M. Morrell, A. Rocha

**Affiliations:** ^1^Clinical Sciences, Swedish University of Agricultural Sciences, Uppsala, Sweden; ^2^Department of Immuno-Physiology and Pharmacology, Instituto de Ciências Biomédicas Abel Salazar, University of Porto, Porto, Portugal; ^3^Centro de Estudos de Ciência Animal, University of Porto, Porto, Portugal

**Keywords:** post-breeding endometritis, acute endometritis, fluid and leucocyte accumulation, bacteria, altered mucociliary activity, plasma cell infiltration, chronic endometritis

## Abstract

One of the most commonly encountered challenges in equine breeding is endometritis, which can be difficult to resolve and causes considerable economic losses to the industry. It is a multifactorial condition, developing as an exaggerated form of the normal physiological response to breeding. Seminal plasma proteins, spermatozoa, bacteria and debris initiate an inflammatory response; the resulting fluid and neutrophils are then cleared from the uterus along with the debris. However, in some mares, the response is prolonged or exaggerated, with much fluid formation and neutrophil infiltration leading to acute endometritis. A bacterial cause has been implicated, although in some cases no pathogenic organisms can be isolated on culture. It has been postulated that any one of a variety of bacteria could be involved, or dysbiosis of the uterine microbiome could be responsible. Repeated episodes of acute endometritis may lead to the pathology associated with chronic endometritis, with mucociliary dysfunction, vascular degeneration and plasma cell infiltration. This review examines the information that is currently available about equine endometritis, particularly about the role of the inseminate in the uterus, and its current treatment. There are some promising lines of research into treatment or prevention that may help to resolve the issue.

## Introduction

**Figure 1:**
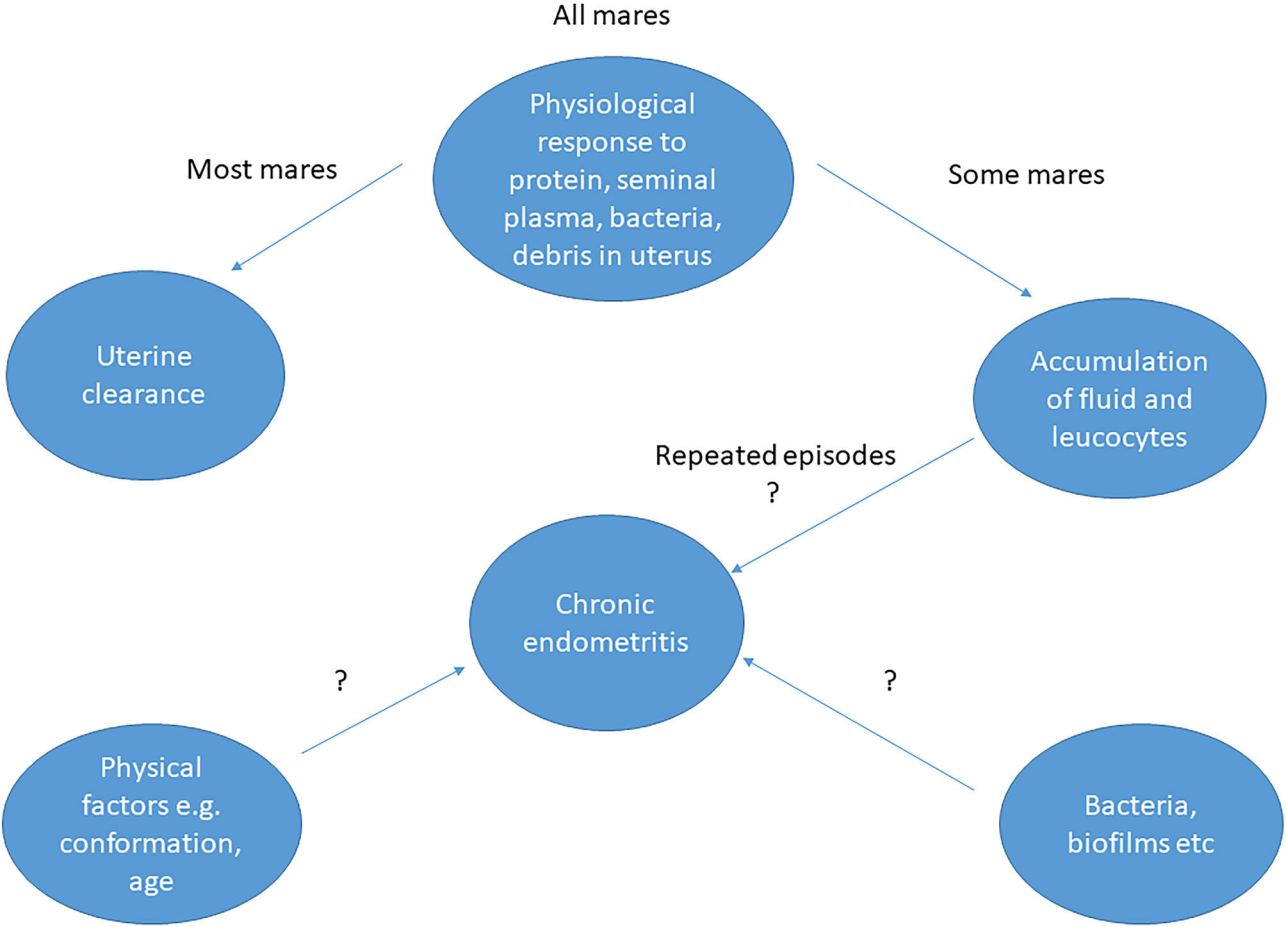
Possible causes of endometritis in mares and interactions between factors. The question marks indicate that the exact cause has not been determined and possible interaction of the various factors is speculative.

Persistent endometritis is one of the most frequent causes of subfertility in equine breeding (1) causing huge economic losses to the equine industry. Despite decades of research, most equine practitioners still use the same methods of treatment for this condition, indicating a need for more effective treatments or non-surgical ways of preventing the condition. The cause of persistent endometritis is multifactorial, ranging from physical factors, such as age and conformation of the perineal area, to an exaggerated post-breeding response of the uterine epithelium (2). Chronic endometritis may arise from repeated episodes of acute endometritis (3). Alternatively, it may be bacterial in origin (Figure 1), together with underlying physical problems relating to the effectiveness of the cervix as a barrier to the entry of microorganisms (4). However, culture of microorganisms may be uninformative; either no organisms are isolated, or the organisms present are not known to be pathogenic. It is not known whether the bacteria isolated are responsible for the condition from the start or are secondary to the underlying cause (3). The fertility issues associated with chronic endometritis may be resolved, at least in some cases, by treatment with antibiotics (5), but such treatment in the absence of a positive identification of a pathogen goes against current guidance on the prudent use of antibiotics. Therefore, a better understanding of persistent endometritis is required in the hope of finding a means of prevention and/or better methods of treatment than are currently available (2).

The purpose of this article is to briefly summarize the information that is currently available about equine endometritis and the effectiveness, or otherwise, of conventional treatments. The components of the inseminate that may contribute to the problem will be examined in more detail. Some new therapies that have emerged in the last decade will be presented together with some promising lines of research for the future. The theory that some cases of chronic endometritis can be prevented by reducing the risk for exaggerated post-breeding endometritis is also discussed.

## Response to Mating and Post-Breeding Endometritis

In all mares, inflammation of the endometrium occurs as a physiological response to mating, mediated by cytokines and complement activation (5). Its function is to clear the uterus of contaminants, seminal plasma and excess spermatozoa before the arrival of the conceptus ~5 days after ovulation (6). Usually fluid and inflammatory cells are removed by uterine contractions within 24–48 h after mating. However, in some mares the fluid and neutrophils are retained for prolonged periods, affecting ciliary function. Altered mucociliary activity allows bacterial adhesion to the endometrium and expulsion of inflammatory cells. Vascular degeneration inhibits hormone delivery to the endometrium and disturbs uterine drainage, reducing venous return to capillary beds (5). Long term chronic degenerative changes in the endometrium result. The role of bacteria in the process is not clear; they are present in semen or in the inseminate and are expelled from the uterus by the immune response just described (7). However, it is not known whether the bacteria are primary or secondary to the pathology occurring in the endometrium (3).

If a mare is seen to be retaining fluid in the uterus after breeding, in some cases it is sufficient to flush the uterus within 48 h after breeding to ensure fluid and neutrophil removal. However, if this inflammation is not halted, a condition known as persistent breeding-induced endometritis ensues, which may lead to chronic endometritis (3). The ability to clear fluid and inflammatory cells from the uterus within 48 h is used to classify mares as being susceptible or resistant to post-breeding induced endometritis (8, 9). Susceptible mares typically show a delay in the initiation of the immune response, which is then prolonged compared with resistant mares (10). The resulting accumulated fluid and debris creates an environment that is hostile to the arriving embryo, and is detrimental to the establishment of pregnancy, perhaps by interfering with the maternal recognition of pregnancy signal (11).

## Chronic Endometritis

Chronic endometritis is multifactorial, with a mare's susceptibility being affected by factors such as age, poor perineal conformation and cervical competence (12), but also potentially by other factors. Microbes are implicated although their presence may be due to the physical factors already mentioned. Bacteria vary in their ability to adhere to epithelia, the viscosity of their secretions, their resistance to phagocytosis, and their ability to induce inflammation (13). However, culture of specific microorganisms from the uterus may be uninformative. Either no organisms are isolated from culture, or the organisms present are not known to be pathogenic, or apparently may be pathogenic in some instances and not in others. Therefore, Koch's postulates are not fulfilled (14). Treatment with antibiotics may result in some females conceiving after treatment (15). Although the uterine microbiome has been characterized in some species, such as the human and cow, little is known about the microbiome of normal mares. In broodmares, persistent endometritis is a frequent cause of sub-fertility (1). A link to the putative introduction of bacteria during breeding is postulated but has not yet been established (5), with the exception of *Taylorella equigenitalis* infection (15) and *Pseudomonas aeruginosa* infection (16). In the latter study, mares were either bred by a stallion known to be infected with *P. aeruginosa* or were inseminated with his semen. More than half of the mares bred by natural mating became positive for the bacterium, whereas 22% of those inseminated had delayed uterine clearance without isolation of *P. aeruginosa*. Therefore, there is circumstantial evidence for the stallion being one of the sources of the bacteria involved in endometritis.

Many cases of persistent endometritis are sub-clinical, manifesting only as a “repeat breeder.” So far, it has not been possible to identify a specific causative agent of chronic equine endometritis. Uterine swabbing and low volume lavage often fail to reveal any causal microorganisms. Clinical signs, such as fluid accumulation in the uterus detected on ultrasound examination, are not reflected in microbiology results; alternatively, in those instances where organisms are identified, they are not known to be pathogenic, although they may be opportunistic in this regard. Treatment is mostly based on antibiotic therapy targeting those bacterial species considered to be pathogenic in the equine reproductive tract (17–19). Although antibiotic treatment in the absence of a positive identification of a pathogen has, in some cases, resolved the fertility problem (20), it goes against current guidance on the prudent use of antibiotics. Antibiotics should only be used where evidence of a bacterial infection exists and after sensitivity testing of the organism concerned. The “One Health” concept, incorporating the prudent use of antibiotics referred to previously, is to restrict the use of antibiotics in cases of endometritis to those in which there is a positive culture (3).

## Diagnosis

The “gold standard” to diagnose persistent equine endometritis is still histopathological evaluation of endometrial biopsies (20, 21) but this technique is rarely used in clinical practice. Common clinical methods such as swabbing or uterine lavage are of low sensitivity (6, 17, 20, 21), with the result that subclinical endometritis is often undiagnosed. Subclinical endometritis has been defined variously by different authors, such as subtle signs of fluid accumulation with the presence of polymorphonuclear leucocytes (PMNs) but an absence of bacteria (6). However, Sikora et al. (22) observed that bacteria could be isolated from some mares in the absence of PMNs, confounding this definition of subclinical endometritis. Overbeck et al. (23, 24) considered mares to have subclinical endometritis if the proportion of PMNs in the cellular content was <2%. There are reports that the sampling technique and its timing could influence the proportion of PMNs (25). In addition, the technique with the highest sensitivity (>80%) to isolate microorganisms, i.e., microbial culture of endometrial biopsies, is infrequently used and may not reveal all organisms present (20, 21, 26). In cattle, Knudsen et al. (27) obtained a more diverse microbiome with biopsy than with uterine flush, and Sicsic et al. (19) reported that failure to cure metritis was associated with a decrease in the diversity of the uterine microbiota. Holyoak et al. (28) performed analysis of the microbiota in the endometrium of mares without clinical signs of endometritis, and concluded that culture-based techniques miss the large diversity that is usually present. The authors speculate that intra-microbiota interactions may be more relevant to maintain the health of the uterus than the presence of any given bacterial species, as has been noted for the cow (29).

Although some of the isolated bacteria are otherwise considered to be non-pathogenic, treatment of the mares with antibiotics may resolve the fertility issue, suggesting that the definition of pathogenic and non-pathogenic bacteria (20) is not infallible. Bacteria that are normally non-pathogenic can cause pathology in some instances if the local environment is favorable for their growth (facultative pathogens). Moreover, it is likely that the pathogens isolated by traditional culture methods represent only the tip of the iceberg. A more effective identification of the whole microbiome present is needed.

It was postulated that endometrial biopsy itself may be beneficial as a treatment in women with chronic endometritis, partly due to the stimulation of growth hormone and cytokine secretion after the artificial injury (30), and partly due to physical removal of bacterial films (31). The claimed improvement in pregnancy rate in women suffering from chronic endometritis was supported by meta-analysis (32, 33). The anatomy and physiology of the mare's endometrium differs considerably from the human uterus, and a pregnancy can be established in mares in the same cycle as a biopsy is performed. However, an experiment to assess a direct beneficial effect of biopsy in mares with chronic endometritis has not been conducted.

## Failure of Antibiotic Therapy

The failure of antibiotic therapy may depend on the chronicity of the infection, the presence of a mixed population of organisms, and whether the infection is focal or diffuse (6). In addition, antimicrobial resistance is increasingly a problem. Several of the organisms commonly isolated from the uterus of mares with endometritis, such as *Pseudomonas, Klebsiella* and *Escherichia coli*, are considered to be highly resistant to antibiotics such as penicillin (34). Antibiotics may not be effective against the biofilm since only the microorganisms on the outside of the biofilm are exposed to their activity. The bacteria most commonly occurring in biofilms are sensitive to different antibiotics, making treatment difficult. Clearly, an alternative method to detect and identify microorganisms in the equine uterus is required, both to provide a more targeted approach to fertility problems and to conform to best practice regarding use of antibiotics. As discussed in section 8, there are anecdotal reports that some non-antibiotic therapies have been successful against organisms in biofilms (20).

Bacteria that grow freely in liquid are said to be planktonic, but some bacteria can also grow on surfaces such as epithelia and endothelia, forming a biofilm (35). Biofilms consist of population(s) of bacteria, which adhere to a surface and to each other and are enclosed in a matrix of biopolymers. The formation of a biofilm starts with bacteria that adhere to a surface, e.g., by means of fimbriae, where they bind irreversibly and initially grow as a monolayer. They then form several layers and start to produce a biopolymer (extracellular matrix) often consisting of the same material as the glycocalyx (capsule), but in a looser structure. The biopolymer is made up of polysaccharides such as dextran. Biofilms consist of one or more bacterial populations, glycocalyx, DNA and proteins. A bacterial species that cannot itself adhere to a surface/epithelia/endothelia, can often become attached to a pre-existing bacterial glycocalyx and grow as a biofilm. Bacteria in biofilms are more resistant to antibiotics, detergents and phagocytosis than planktonic bacteria. Microbes such as *P. aeruginosa, E. coli* and *K. pneumoniae* and even *Streptococcus zooepidemicus* (3) can form biofilms which protect the organisms from leucocytes and also prevent penetration of topical antibiotics (18). Thus, they are difficult to remove completely from the uterus.

Fungal endometritis can arise from dysbiosis due to previous attempts to treat bacterial infections (36, 37). They are believed to account for 1–5% of endometritis cases, either combined with bacteria or by themselves. Aspergillus and Candida are the organisms most frequently encountered (38).

## The Uterine Microbiome

Several studies have been carried out on the uterine microbiome of human patients, but the relevance of the findings to breeding animals is unknown. Among livestock species, most of the studies on uterine microbiota have been conducted in cattle. In a comparison of the uterine microbiome in healthy cows and those with either clinical or subclinical endometritis (13, 9, and 5 animals, respectively) the diversity of the flora was similar between the groups (39). However, the uterine microbiota of cows with clinical endometritis contained an increased abundance of Fusobacterium; furthermore, *Trueperella* and Peptoniphilus were found only in these cows. In cows with endometritis, *Lactobacillus* and *Acinetobacter* were present, although no known pathogens were identified. Pascottini et al. (40) reported differences in the microbiome of cows with endometritis, with a loss of diversity and an increased prevalence of *Trueperella pyogenes*. There were no differences between healthy cows and those with subclinical endometritis. The number of cows in the latter study was 5 with clinical endometritis, 8 each in the healthy and subclinical endometritis groups. However, in another study, the uterus of virgin heifers and pregnant cows was found to contain several bacteria commonly associated with postpartum pathology, including Trueperella and Fusobacteria (41). These authors speculate that the uterine microbiome is already present by the time heifers reach maturity and that pregnancy is maintained in its presence. It seems that the intricacies of microbial interactions are important in the establishment or prevention of uterine pathology (39). It is also clear that 16S sequencing facilitates the identification of the entire microbial spectrum, whereas conventional culture and identification techniques will reveal only a part of the microbiome.

According to Jones (42) the most frequent phyla in the uterus and vagina of 16 healthy mares were Bacteroidetes, Firmicutes, Actinobacteria, Proteobacteria, and Verrucomicrobia. The dominant genus in the equine uterus was Corynebacterium, followed by Porphyromonas, Enterobacteriaceae, and Streptococcus.

## Role of Seminal Components and Semen Extender in Persistent Endometritis

The presence of seminal components in the uterus invokes a complement cascade in uterine secretions, mediating vascular permeability and phagocytosis (1). Macrophages and PMNs attracted by chemotaxis become activated, extruding DNA to form neutrophil extracellular traps that engulf bacteria and dead spermatozoa. Many studies have been performed in an attempt to elucidate the role of spermatozoa, seminal plasma and semen extender in this process but the results (and their interpretation) differ. Previously it was thought that seminal plasma was an essential component to protect spermatozoa from the uterine immune response (1). However, Portus et al. (43) studied uterine contractility and PMNS in 41 susceptible mares following insemination with sperm samples in either skimmed milk extender or seminal plasma. At 6 h after insemination, uterine lavage contained more PMNS in the seminal plasma group than the skimmed-milk group, whereas uterine contractility was decreased. Pregnancy rates were similar between treatments. These results suggest that uterine clearance might be hampered in the presence of seminal plasma in susceptible mares. Therefore, it might be best to avoid seminal plasma.

In an experiment to determine the influence of semen extender and seminal plasma on the uterine response to insemination (44), 8 mares were each inseminated in subsequent cycles with phosphate buffered saline, skimmed milk extender, egg-yolk extender or seminal plasma. All inseminations produced an inflammatory response in terms of increased endometrial cell expression of interleukin 1b, interleukin 6, tumor necrosis factor-α and cyclooxgygenase-2 (COX-2). However, seminal plasma stimulated higher COX-2 mRNA expression than PBS, while egg yolk extender provoked the least response.

Further weight to the suggestion that seminal plasma may not be essential to protect spermatozoa in the uterine environment arises from the fact that pregnancies occur following inseminations with frozen-thawed sperm samples, in which most of the seminal plasma is removed before freezing (45). This observation tends to suggest that presence of seminal plasma is not essential or that very little is required to produce a protective effect. The increased pregnancy rates following insemination with colloid-selected spermatozoa, in which seminal plasma is removed (46), also contribute to the argument that seminal plasma is not necessary in certain circumstances, at least for ejaculated spermatozoa. However, the uterine response may be different in mares susceptible to persistent post-breeding endometritis.

One explanation for the differing opinions as to whether seminal plasma is required or not could be that in some studies spermatozoa are washed, purportedly to remove seminal plasma. However, in a study with boar spermatozoa, washing by centrifugation did not remove the seminal plasma proteins coating the spermatozoa; only colloid centrifugation removed these proteins (46). Therefore, it is problematic to interpret the results of insemination studies in mares with “washed” spermatozoa, unless complete removal of seminal plasma is shown e.g., by seminal plasma markers. In a study in which colloid centrifugation was used to prepare donkey spermatozoa free of seminal plasma, there was increased binding of spermatozoa to PMNs (47). Two specific fractions of seminal plasma were responsible for these effects: proteins in the ranges of 30–50 kDa and 50–100 kDa had a beneficial effect on sperm motility, membrane integrity and binding to PMNs whereas smaller proteins had either no effect or a detrimental effect on these parameters (48). Although sperm binding to PMNs might suggest that these spermatozoa are then lost from the uterus, other studies showed that most of the viable spermatozoa bound to PMNs are not engulfed by them but are subsequently released, and are thus available to proceed further into the female reproductive tract (48). Since the role of PMNs is to engulf bacteria and dead or dying spermatozoa, the interaction between spermatozoa and PMNs may play an important role in the sperm selection mechanisms of the female. If the increased binding of colloid-selected spermatozoa to PMNs is followed by their release, it could help explain the improved pregnancy rate following insemination of colloid-prepared stallion sperm samples (49), apart from its role in reducing the numbers of dead or damaged spermatozoa prior to insemination.

Several studies were conducted to characterize the uterine response to seminal plasma proteins and to clarify their role in endometritis. Seminal plasma proteins were suggested by some researchers to provoke the immune response whilst others have reported that these proteins protect spermatozoa from attack by neutrophils (50). However, in a study to define the role of particular proteins in seminal plasma, CRISP-3 and lactoferrin were reported to have a minimal effect on expression of endometrial cytokines 6 h after insemination in 6 mares (51). Similarly, adding seminal plasma to frozen-thawed sperm samples at artificial insemination did not influence post breeding uterine inflammation or pregnancy rate in 15 healthy mares (52). Sperm motility, judged subjectively, was considered to be lower in the presence of added seminal plasma. This result is in contrast to other studies where adding seminal plasma to fresh spermatozoa increased sperm motility (53, 54).

Another clue to the reason for differing opinions on the role of seminal plasma may come from a previous study on the effects of stallion seminal plasma on spermatozoa *in vitro*, where considerable differences between seminal plasma from different stallions was observed (55). The combination of each seminal plasma sample and sperm sample provided a unique set of conditions with a unique outcome in terms of sperm motility and chromatin damage. If this difference between individual samples extends to interactions within the uterus, it could help to explain the diversity of results obtained in studies on the effects of seminal plasma on the uterine response. Presumably each seminal plasma sample evokes a unique response in the mare depending on the combination of seminal plasma factors, uterine factors and sperm factors.

## Treatment Options

The conventional methods to treat equine endometritis involve uterine lavage, with or without ecbolics such as oxytocin or prostaglandins, to aid uterine clearance, antibiotics (systemic or local), with vulvoplasty to correct conformational issues (56). These treatments have been available for decades and are well-reported in the literature, but the persistence of the problem in equine breeding suggests that new approaches might be necessary, perhaps in combination with some of the conventional therapies. A number of non-traditional therapies have been proposed, although some of them are based on small sample sizes (57). These approaches, including antimicrobial peptides, immunotherapy, platelet-rich plasma (PRP) and stem cells were thoroughly reviewed by Scoggin (57) and will be mentioned only briefly here. Of these, the most promising would appear to be PRP.

### Antimicrobial Peptides

Peptides are released by neutrophils and exert an antibacterial effect (58). A biomimetic substance, Ceragyn, is reported to be active against most of the important organisms implicated in equine endometritis, namely both free-floating and biofilms of *S. equi* ssp *zooepidemicus, E coli*, and *K. pneumoniae*, although apparently the activity against Pseudomonas was less convincing (57). There is a suggestion that bacteria do not become resistant to these substances although this speculation remains to be substantiated (58).

### Immunotherapy

The immune system can be modulated or stimulated by various substances. Glucocorticoids, platelet-rich plasma and stem cells are considered to modulate the immune response whereas mycobacterial cell wall extract stimulate it e.g., to reduce persistent inflammation.

#### Glucocorticoids and Mycobacterial Wall Extract

In a small study (6 mares), insemination with control killed sperm samples in extender or the same sperm samples supplemented with either dexamethasone or mycobacterial wall extract was conducted in consecutive cycles (59). Endometrial biopsies were taken 6 h after insemination. The treatments were associated with a decrease in expression of IL1β mRNA compared to controls. The expression of interleukins (IL) 1RA, 6 and 10 were not affected, although the difference in interferon γ mRNA expression was reported to approach significance. However, the effect of these treatments in conjunction with insemination of viable spermatozoa was not reported. Previously, systemic administration of prednisolone increased conception rate in mares prone to persistent endometritis when inseminated with frozen thawed semen (60), although the mechanism of action was not studied.

#### Platelet-Rich Plasma

Platelet-rich plasma, with its high concentration of growth factors having a mitogenic and anti-inflammatory effect, is considered to be an emerging therapeutic application in tissue regeneration (61). Platelet-rich plasma reduced cyclooxygenase 2 (COX-2) levels in 13 mares susceptible to developing persistent endometritis. Treatment was associated with an increased conception rate in these mares, regardless of whether it was administered 24 h prior to AI or 4 h after AI (62). In a larger study, 73 Arabian repeat-breeder mares were treated with either PRP or lyophilised growth factors from horse platelets (L-GFEquina). The length of the oestrous cycle was shortened, endometrial thickness was increased and pregnancy rate was increased in the treated mares (63).

#### Stem Cells

Mesenchymal stem cells derived from equine adipose tissue were administered into the uterus of 6 mares with varying degrees of endometrosis (64). After 7 and 21 days, the cells were observed to be clustered in periglandular and glandular tissues, suggesting proliferation in all but the most severely affected cases. Although minimally invasive for the mare, the technique is technically demanding in terms of cell culture. The effects of treatment on subsequent reproductive performance have not been tested.

Stem cells derived from bone marrow were reported to have a beneficial effect on endometrial architecture in 16 mares but subsequent reproductive performance was not reported (65). Again, the production of stem cells requires specialist culture techniques.

### Treatment of Equine Endometritis in Practice

Despite the existence of a variety of treatment options (Table 1), evidence-based reports on their effectiveness are lacking (66). A recent survey of equine practitioners in Germany concluded that most still mainly use systemic antibiotic therapy for equine endometritis (67). Slightly less than one half of respondents used uterine lavage in addition to the systemic antibiotics. For uterine lavages, practitioners had mostly changed to 0.9% saline instead of potentially more irritant substances such as povidone iodine, N-acetylcysteine, hydrogen peroxide, dimethyl sulfoxide, or kerosene. Köhne et al. (67) called for more research and better dissemination of results to clinicians in an effort to provide effective treatment.

**Table 1:** Summary of treatment options for equine endometritis.

**Treatment**	**Used for**	**Advantages**	**Disadvantages**	**Comments**
Vulvoplasty	(Temporary) resolution of some conformational defects	Prevents ingress of bacteria; may hinder development of chronic endometritis	Must be reversed prior to foaling or mating and redone afterwards	Prevents bacterial infection of the uterus; has welfare/ethical implications
Uterine lavage +/- ecbolics	Removes excessive fluid & inflammatory cells	Creates better uterine environment prior to arrival of embryo	May not prevent development of chronic endometritis	Symptomatic only; requires monitoring of mare
Antibiotics (systemic or local)	Treatment of bacterial infection	Kills bacteria causing problem	Kills other bacteria too. May predispose to dysbiosis.	Do not always have a positive culture from uterus. May encourage development of antimicrobial resistance; may go against “prudent use” recommendations
Disruptors of biofilms	Anti-biofilms	May remove biofilm	May not be effective; some are irritant to uterine tissue	Many practitioners use saline now
Antimicrobial peptides	Attacks bacteria	“natural” antibacterial effect	Bacteria may become resistant (opinions differ)	No independent controlled studies
Immunotherapy: glucocorticoids, mycobacterial cell wall extract, platelet-rich plasma, stem cells	Modulate or stimulate the immune system	Some potentially interesting results in mares susceptible to acute endometritis	Glucocorticoids may not be effective in chronic cases. Apart from glucocorticoids, technically demanding to produce cells; can be expensive	No large controlled studies; effect in chronic cases uncertain
Identification of microbiota in healthy and susceptible mares	To aid understanding of dysbiosis	Could facilitate choice of suitable mares for breeding	There may not be a difference between healthy and susceptible mares	No large scale studies yet
Develop new semen extenders	Inseminate	Alternatives to protein avoid stimulating immune response	May not sustain stallion spermatozoa	Not reported yet: could prevent chronic cases by reducing acute response
Colloid centrifugation	Removal of all seminal plasma from inseminate	Improves sperm survival; increases pregnancy rate in normal mares; reduces fluid production in susceptible mares	None known	Not reported yet: could prevent chronic cases by reducing acute response if used for all semen doses in maiden mares

*Conventional treatments are shown in black; novel therapies are shown in blue*.

## Future Possibilities

Due to the multifactorial nature of the condition, there are several routes for potential solutions to this challenge. These range from complete identification of the microbiome to development of semen extenders that do not contain protein, and alternative ways of processing semen. Some of these possibilities are considered here.

### Further Identification of the Uterine Microbiome

Sequence-based technology, i.e., metagenomics, offers an alternative to conventional culture and identification of microorganisms. This method is not dependent on being able to culture organisms but instead identifies particular gene sequences, which can then be compared with known sequences to provide an identification with a specified degree of precision. All bacteria possess a specific region known as the 16S rRNA gene, which contains a high number of hypervariable regions; this gene has been used as a major identifier of bacteria (68), enabling bacterial DNA to be distinguished from host DNA. The advantage of this method is that even bacteria that cannot be cultured, either because they are dead or because the culture conditions are not correct, or even because their growth is masked by other species, can be identified due to the presence of the 16S sequence. Thus, Al-Kass et al. (69) identified 83 bacterial types in stallion semen using 16S sequencing, considerably more than the number of species found by conventional culture (70, 71). Identification of the uterine microbiota using the same technique could help to identify a specific microbial cause of persistent endometritis, thus facilitating the search for an effective treatment. Although the uterine microbiome of healthy mares was studied by Jones (42), a similar study on susceptible mares in the same environment is needed.

### Disruption of Quorum Sensing

Biofilm formation is regulated by quorum sensing (72). The latter is a system for signaling and response in a population of organisms (e.g., bacteria), regulated by the population size. In other words, the gene expression of certain proteins can be regulated in response to changes in population density. Synthesis of the constituents of a biofilm is initiated when the bacterial population density has reached a certain level. Possibilities for treating a biofilm could be to disrupt their quorum sensing ability or to attack the extracellular matrix holding them together. Ceragyn, hydrogen peroxide, acetylcysteine and EDTA-Tris are some of the most frequently utilized chemicals to treat endometritis caused by potentially biofilm-producing bacteria (57), but controlled studies on their efficacy are lacking. Several plant-based substances are active in this regard and could be potential methods of attack, e.g., curcumin (72).The efficacy of these substances should be investigated, providing that they do not damage the uterine epithelium.

### Alternatives to Proteins in Semen Extenders

Almost all of the semen extenders used in equine breeding contain protein of some description, either milk-based e.g., Kenney's extender (73), either home-made or a commercial variant, or egg-yolk e.g., Ghent extender. In other species it has been possible to replace proteins of animal origin with synthetic substances e.g., polyvinylalcohol for some boar semen extenders (Kiev extender) (74), or liposomes for bull semen (Optixcell, IMV Technologies) (75). Therefore, it would be interesting to investigate whether similar formulations would support stallion spermatozoa whilst potentially not stimulating an exaggerated response from the uterine epithelium.

### Avoidance of Seminal Plasma in Insemination Doses

There is some debate about the precise role of seminal plasma in the post-breeding inflammatory response, as discussed in section 7; it may be one of the drivers provoking the condition or may be a modulator of this response. A PMN influx into the uterus occurs in response to seminal plasma (76, 77), although the duration of the inflammatory response is then shorter than in the absence of seminal plasma (77, 78). Other theories favor involvement of seminal plasma in protecting spermatozoa from binding to PMNs and being phagocytosed; CRISP3 protein in seminal plasma inhibited the binding mechanism between viable spermatozoa and PMN involved in phagocytosis (79). These findings, therefore, lead to the question of whether seminal plasma should be removed when preparing insemination doses. In some countries it is customary to remove most of the seminal plasma by centrifugation when preparing insemination doses, in the belief that this will prolong sperm survival during storage. However, such centrifugation may damage sperm chromatin, particularly if high centrifugal force is used. Furthermore, sperm washing does not remove the seminal plasma proteins coating the sperm surface, unlike colloid centrifugation that does remove such proteins (46). However, centrifugation through a colloid e.g., Single Layer Centrifugation (SLC) selects the most robust spermatozoa from the ejaculate for artificial insemination and can improve pregnancy rates for problem stallions (80). Pregnancy rates may be improved even for stallions with normal pregnancy rates (49), probably due to removal of spermatozoa with damaged chromatin. The selected spermatozoa can be stored for prolonged periods and retain their fertilizing ability; in a pilot study, SLC-selected sperm samples stored for up to 96 h before insemination produced the same number of conceptions as would normally be expected after 24 h storage (81). These results suggest that seminal plasma is not a pre-requisite for normal fertility, at least in the majority of mares. There is always the possibility that the situation is different in susceptible mares.

In a small insemination study involving six mares known to produce an exaggerated response following artificial insemination, the semen was prepared by SLC immediately before insemination. None of the mares showed an accumulation of fluid afterwards; a small volume of fluid was seen in one of the mares but was not deemed to be sufficient to warrant flushing. Three of the mares conceived (J Grossman and JM Morrell, unpublished data), despite not conceiving after insemination in many cycles in previous years. Unfortunately, it was not possible to carry out a controlled trial, i.e., inseminations with SLC-selected semen alternating with inseminations with control semen from the same stallion in different cycles in the same mares, since the object of the insemination in these cases was to establish a pregnancy. However, such a controlled trial would be worthwhile to provide proof of principle of this theory, if suitable mares could be found.

In a recent study with donkey spermatozoa and PMN, enhanced binding to PMN was observed for the SLC-selected sperm samples compared to controls (47). These results are in agreement with previous studies, which showed that CRISP3 protein in seminal plasma inhibited the binding mechanism between viable spermatozoa and PMNs involved in phagocytosis (79). Since insemination of SLC-selected sperm samples results in a higher pregnancy rate than control samples (49), it can be speculated that the removal of these seminal plasma proteins from the sperm surface by colloid centrifugation facilitates selective binding of certain spermatozoa by PMN and hence the removal of spermatozoa with undesirable characteristics from the uterus. Thus, the ability of the reproductive tract to permit only certain spermatozoa to reach the oviducts would be enhanced and fertility improved.

Although promising as a means of preventing the exaggerated response to breeding in some mares, the small study on SLC described above does not reveal the actual cause. It was interesting to note, however, that spermatozoa alone did not invoke the exaggerated response, after they have passed though the colloid. Colloid centrifugation removes most of the bacteria from the semen sample (82, 83) as well as seminal plasma proteins. Thus, it is not known whether it is removal of seminal plasma *per se* that is beneficial or the removal of the bacteria contained in the seminal plasma. The ejaculate of healthy stallions contains bacteria that colonize the mucosa of the reproductive tract and are transferred to the semen during ejaculation. The response of the uterus following insemination is partly to inactivate these bacteria and ensure their removal, as previously explained. Since the majority of the bacteria are environmental in origin, there is little to be done to prevent their appearance in semen other than to keep stallions in a clean environment and use strict hygienic conditions when collecting and handling semen. Adding antibiotics to the semen extender may kill most of the bacteria but does not remove them from the sample. Since dead bacteria still elicit the immune response, it would seem to be prudent to remove as many bacteria as possible from the sample immediately after semen collection rather than to add antibiotics in an attempt to kill them.

It should be noted, however, that removing the seminal plasma from insemination doses by colloid centrifugation will not prevent the development of endometritis due to poor conformation of the perineal area (2). Such conformational problems would be apparent during a breeding soundness examination, during which the suitability of the mare for breeding is assessed. Although vulvoplasty has been used as a preventative measure, it can result in some mares being used for breeding that would otherwise be unable to conceive because of poor conformation. In cases where poor conformation is likely to be an inherited condition, the owner should be advised not to use such an individual for breeding.

### Combinations of Various Alternatives

The possibilities listed previously are not restricted to individual use; they could be tried in different combinations, such as colloid centrifugation in combination with modified extenders. In addition, strict hygienic precautions should be maintained at all times when inseminating mares or carrying out any reproductive examinations, and only individuals that are suitable for breeding should be chosen as broodmares.

## Conclusions

Despite decades of research, the exact cause of acute and chronic endometritis in mares is still unknown. A link between acute and chronic endometritis is suggested but is not proven; similarly, a bacterial cause is assumed but does not fit Koch's postulates. It is always difficult to draw proper conclusions based on *ad hoc* observations or small clinical trials lacking in power. Future research into methods of treating biofilms could prove to be rewarding but it could be beneficial to avoid the problem altogether. Novel semen extenders that do not contain protein could be useful but probably the most effective way of avoiding the problem would be to remove all seminal plasma (and most of its load of bacteria) from semen doses for artificial insemination by colloid centrifugation.

## Data Availability Statement

The original contributions presented in the study are included in the article/supplementary material, further inquiries can be directed to the corresponding author/s.

## Author Contributions

All authors listed have made a substantial, direct, and intellectual contribution to the work and approved it for publication.

## Funding

JM is funded by the Veterinary Faculty, Swedish University of Agricultural Sciences.

## Conflict of Interest

JM is the inventor and one of the patent holders of the colloid mentioned in this review. The remaining author declares that the research was conducted in the absence of any commercial or financial relationships that could be construed as a potential conflict of interest.

## Publisher's Note

All claims expressed in this article are solely those of the authors and do not necessarily represent those of their affiliated organizations, or those of the publisher, the editors and the reviewers. Any product that may be evaluated in this article, or claim that may be made by its manufacturer, is not guaranteed or endorsed by the publisher.
